# Consensus on the tertiary prevention of primary liver cancer

**DOI:** 10.1007/s12072-023-10549-2

**Published:** 2023-06-27

**Authors:** Yuemin Nan, Xiaoyuan Xu, Shiming Dong, Ming Yang, Ling Li, Suxian Zhao, Zhongping Duan, Jidong Jia, Lai Wei, Hui Zhuang, Chinese Society of Hepatology, Chinese Medical Association, Hongsong Chen, Huiguo Ding, Zhongping Duan, Jian-gao Fan, Yanhang Gao, He-ping Hu, Jianrong Huang, Jun Li, Wencong Li, Wen-gang Li, Jingfeng Liu, Lingdi Liu, Yuemin Nan, Wanhua Ren, Jia Shang, Maorong Wang, Wen Xie, Mengsu Zeng, Yuguo Zhang, Jingmin Zhao, Shousong Zhao, Weifeng Zhao, Jian Zhou

**Affiliations:** 1 Department of Traditional and Western Medical Hepatology, The Third Hospital of Hebei Medical University, Shijiazhuang, 050051 China; 2 Department of Infectious Diseases, Peking University First Hospital, Beijing, 100034 China; 3 Peking University People’s Hospital, Peking University Hepatology Institute, Beijing, China; 4 Department of Intervention, Mengchao Hepatobiliary Hospital of Fujian Medical University, Fuzhou, 350025 China; 5 Artificial Liver Centre, Beijing You-An Hospital, Capital Medical University, Beijing, 100069 China; 6 Liver Research Centre, Beijing Friendship Hospital, Capital Medical University, Beijing, 100050 China; 7 Hepatopancreatobiliary Centre, Beijing Tsinghua Changgung Hospital, Tsinghua University, Beijing, 102218 China; 8 Department of Microbiology and Centre for Infectious Diseases, Peking University Health Science Centre, Beijing, 100191 China

**Keywords:** Hepatocellular carcinoma, Tertiary prevention, Curative treatment, Recurrence, Metastasis, Risk stratification, Surveillance, Detection, Diagnosis, Survival

## Abstract

To effectively prevent recurrence, improve the prognosis and increase the survival rate of primary liver cancer (PLC) patients with radical cure, the Chinese Society of Hepatology, Chinese Medical Association, invited clinical experts and methodologists to develop the *Consensus on the Tertiary Prevention of Primary Liver Cancer*, which was based on the clinical and scientific advances on the risk factors, histopathology, imaging finding, clinical manifestation, and prevention of recurrence of PLC. The purpose is to provide a current basis for the prevention, surveillance, early detection and diagnosis, and the effective measures of PLC recurrence.

## Introduction

Primary liver cancer is one of the common malignant tumors and the main causes of tumor death. The main pathological types include hepatocellular carcinoma (HCC), intrahepatic cholangiocarcinoma (ICC), combined HCC–ICC and rarely undifferentiated liver cancer. Of those, HCC accounts for 75– 85% [1]. In this Consensus, “primary liver cancer” mainly refers to HCC.

In a broad sense, the tertiary prevention of primary liver cancer refers to the use of measures to improve the survival rate and life quality of patients by taking effective anti-tumor, anti-recurrence and metastasis, etiologically related disease treatment and recurrence surveillance in the population with a confirmed diagnosis of HCC. With the continuous improvement of diagnosis and treatment of liver cancer, the neoadjuvant therapy and conversion therapy have significantly improved the surgery and local ablation therapy rate of HCC, and the population with access to curative treatment is growing. In this Consensus, tertiary prevention of primary liver cancer is the strategy to further reduce recurrence rate and mortality and improve the overall survival in patients with HCC following curative treatment.

According to the Global Cancer Statistics, Globocan 2020 [1], there were 906,000 new cases of liver cancer, with the incidence ranking sixth in malignant tumors, and 830,000 deaths, with the mortality ranking third. There were 657,000 new cases and 609,000 deaths in Asia, accounting for 72.5% and 73.3% worldwide. Although the incidence and mortality rates of liver cancer have decreased in many high-risk countries and regions in Eastern and South-Eastern Asia, the highest liver cancer death-to-incidence ratios were presented in Southeast, Southern, Central and Western Asia. In China, there were 410,000 new cases, ranking fifth in malignant tumors, and 391,000 deaths, with the mortality ranking second. Due to differences of the regions, management and policies, there are significant differences in the 5-year survival rate of liver cancer, which is only 11.7–14.1% in China [2]. Reducing the recurrence rate of liver cancer after curative treatment and improving the early diagnosis rate of recurrent liver cancer are important measures to improve the 5-year survival rate.

## The consensus development process

In November 2021, the Chinese Society of Hepatology, Chinese Medical Association issued *Consensus on the Secondary Prevention of Primary Liver Cancer* [3]. A panel of experts consisting clinical epidemiologists, hepatologists, hepatobiliary surgeons,interventional radiologists, and oncologists, were organized to formulate the *Consensus on Tertiary Prevention of Primary Liver Cancer* based on the current scientific evidence and practicing norms in the risk factors, pathological mechanisms, preventive measures, surveillance and diagnostic techniques, and related treatment of HCC recurrence in the clinical practice in the Asia–Pacific region and worldwide. The contents of the Consensus have been refined by the panel through multiple rounds of discussions, debates, and revisions. The recommendations for the controversial issues were generated through voting and only those with more than two-thirds of the votes cast were reserved.

This Consensus is formulated in accordance with the principles of evidence-based medicine as listed in the Grading of Recommendations Assessment, Development, and Evaluation System (GRADE). The Consensus designates the quality of evidence by one of three levels: A, B and C and classifies the strength of recommendations as strong (1) and weak (2), as shown in Table 1. The Consensus aims to provide clinicians in liver disease and related specialties with reasonable suggestions and decision-making references for the surveillance, diagnosis and prevention of recurrence after curative treatment of liver cancer. This Consensus will be continuously updated and improved according to the latest clinical medical evidence.

**Table 1:** Quality of evidence and grades of strength of recommendations of the GRADE system

Grade	Description
Quality of evidence	
High (A)	Further research is unlikely to change our confidence in the estimate of effect
Moderate (B)	Further research is likely to have an impact on our confidence in the estimate of effect and may change the estimate
Low or very low (C)	Further research is very likely to have an impact on our confidence in the estimate of effect and is very likely to change the estimate
Strength of recommendation	
Strong (1)	It is clearly demonstrated that the benefits outweigh the risks or vice versa
Weak (2)	Benefits and risks are uncertain, or are demonstrated to be balanced irrespective of the evidence quality

## Terminology

**Fig. 1:**
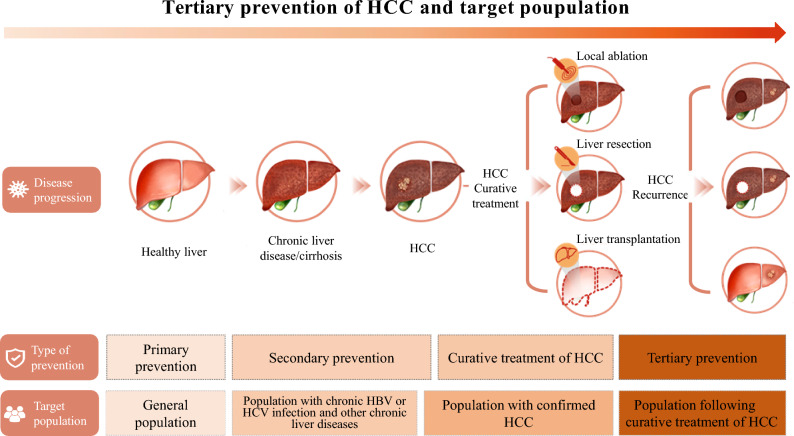
Flowchart for tertiary prevention of HCC

**Tertiary prevention of primary liver cancer**: the comprehensive measures adopted in the population following curative treatment of HCC to surveil and control the etiologically related diseases and prevent recurrence and metastasis of HCC according to the recurrence risk stratification, so as to reduce the recurrence of HCC, improve the early diagnosis rate of recurrent HCC and the long-term prognosis (Fig. 1).

### Curative treatment of HCC

The management for complete elimination of HCC tumor lesions including liver resection, liver transplantation, local ablation and stereotactic body radiation therapy (SBRT), by which the following goals can be achieved: absence of cancer cells or cancer tissue residues, no vascular and bile duct invasion under microscopic observation of the liver resection margin, and no evidence of lymph node or extrahepatic distant metastasis; no HCC characteristics by imageological and serological examination after treatment for two months. Curatively treatable HCC stages include stages 0–A according to Barcelona clinic liver cancer (BCLC) staging system or stages Ia–IIa according to the China liver cancer (CNLC) staging system.

### Microvascular invasion (MVI)

Presence of cancer cell clusters in the vascular lumen lined by endothelial cells under a microscope in the histopathological observation of the liver. According to pathological features, it is divided into: M0, no MVI found; M1, low risk MVI, ≤ 5 MVI within 1 cm in para-cancerous liver tissue; M2, high risk MVI, > 5 MVI within 1 cm in para-cancerous liver tissue or MVI in distant para-cancerous liver tissue (> 1 cm).

### HCC recurrence

HCC recurrence after curative treatment, which, according to the time to recurrence (TTR), can be divided into short-term recurrence (TTR < 2 years) and long-term recurrence (TTR ≥ 2 years), and divided into intrahepatic recurrence, extrahepatic metastasis and intrahepatic recurrence with extrahepatic metastasis by site of recurrence and metastasis.

### Intrahepatic recurrence (IHR)

IHR, referring to the appearance of new HCC lesions in the liver after curative treatment of HCC, is divided into intrahepatic metastasis (IM) and multicentric occurrence (MO). IM, derived from primary HCC, with recurrent tumors equally or less differentiated than primary tumors, is more common in moderately/poorly differentiated HCC; MO is recurrence of HCC on the basis of progressive liver disease, with no definite relation with primary HCC.

### Extrahepatic metastasis (EHM)

After curative treatment of HCC, tumor cells continue to grow into metastatic tumors in other tissues and organs through blood flow or lymphatic circulation. Hematogenous metastasis is more common in the lung, kidney, adrenal gland, bone and brain. Lymphatic metastasis is seen in the hilum of liver, hilum of spleen, parapancreatic, para-aortic and supraclavicular lymph nodes. Infiltration and implantation metastasis can be seen in the diaphragm, pleura, peritoneum and ovary.

### Routine surveillance for HCC recurrence

An approach to surveil and diagnose HCC recurrence using serum alpha-fetoprotein (AFP) or combined with lens culinaris agglutinin-reactive fraction of AFP (AFP-L3), des-gamma-carboxy prothrombin (DCP)/protein induced by vitamin K absence or antagonist II (PIVKA-II) and routine abdominal ultrasound, or liver multiparametric magnetic resonance imaging (MRI), dynamic contrast-enhanced computed tomography (CT) imaging.

### Enhanced surveillance for HCC recurrence

Use of multiparametric MRI alternating combined with CT dynamic contrast-enhanced imaging to surveil HCC intrahepatic recurrence and extrahepatic metastasis, and if necessary, combined with positron emission tomography (PET)-CT and/or bone scan, including the surveillance of target organs such as liver, lung, adrenal gland, lymph node and bone.

### Recurrence-free survival (RFS)

Time from curative treatment of HCC to tumor recurrence or death from any other cause.

## Recurrence rate of HCC following the curative treatments

According to global multicenter data, the 5-year recurrence rate after liver resection is as high as 40–70%, mainly IHR [4, 5], and the 5-year survival rate after re-resection following recurrence is about 30–40% [6, 7]. The 5-year recurrence rate after local ablation therapy is about 50–70% [8], and EHM often occurs after re-treatment of IHR. The 10-year recurrence rate after liver transplantation is 10–15% [8], and the recurrence pattern is mainly EHM, with lung metastasis accounting for 38% [9].

The 5-year survival rates of patients with and without recurrence were 23% and 47%, respectively. The survival of patients was reduced by about 54 months with recurrence [10]. Therefore, after the primary curative treatment for HCC, strict surveillance, effective prevention and early detection of HCC recurrence should be carried out.

- Recommendation 1: HCC recurrence is increasing with clinical stage progression (BCLC 0–A/CNLC Ia–IIa). The short-term recurrence is mainly IHR. The 5–10-year cumulative recurrence rate increases with time, and effective measures should be taken to prevent and surveil recurrence after curative treatment of HCC (A, 1).

## Risk factors for HCC recurrence after curative treatment

### Morphological and pathological characteristics of HCC

Invasive pathological features of HCC are associated with short-term recurrence after curative treatment. Tumor diameter > 5 cm, large number, no capsule or incomplete capsule, poorly differentiated tumor cells, MVI and satellite lesions are risk factors for recurrence. After liver resection, the risks of EHM in patients with tumor diameter 3–5 cm and > 5 cm were 2.86 and 4.72 times of those with tumor diameter < 3 cm, respectively [11]. The recurrence rate of HCC patients with 2–3 tumors was significantly higher than that in patients with single tumor [12, 13]. Patients with capsular invasion of HCC tumors had a risk of HCC recurrence 2.06 times higher than that in patients with intact capsules [14]. Primary HCC with poorly differentiated tumor cells had a higher risk of HCC recurrence after liver transplantation.

MVI of portal vein branch is a potential source of intrahepatic metastasis, and MVI of hepatic venous system is a major source of postoperative recurrence and distant metastasis. Radiomics based on imaging modalities had a comparable identification performance for the preoperative HCC–MVI status, in which sensitivity and specificity were 84% and 83% [15]. For HCC with MVI (compared with HCC without MVI), the recurrence rate increased to 3.1 times at 1 year after liver resection and 3.9 times at 5 years after liver transplantation [16]. The incidence of MVI was significantly higher in patients with single tumor size > 5 cm and patients with 2–3 tumor nodules [17].

Satellite lesions around HCC tumor are mainly caused by intrahepatic metastasis, which can indicate tumor aggressiveness. The incidence of satellite lesions was significantly higher in patients with single tumor > 3 cm than that in patients with single tumor ≤ 3 cm (35.5% vs 12.3%) [18]; the 5-year recurrence rates with and without satellite lesions were 37.5% and 16.8%, respectively.

### Serum HCC biomarker levels

Serum AFP, AFP-L3 and PIVKA II/DCP level before curative treatment of HCC as well as post-treatment normalization can reflect the invasiveness of tumors to some extent and predict the risk of recurrence.

#### AFP

As a routine indicator for early warning and diagnosis of HCC, AFP level before curative treatment as well as post-treatment normalization can predict the risk of recurrence of HCC. AFP ≥ 200 ng/mL is an independent predictor of MVI. Compared between patients with AFP ≥ 200 ng/mL and < 200 ng/mL, the incidence of MVI (40.0% vs 17.8%), poorly differentiated tumor cells (WHO G3, 11.1% vs 3.1%), and EHM risk (3.16:1) were significantly increased. Stratified by AFP < 20 ng/mL, 20–400 ng/mL and > 400 ng/mL, the 2-year recurrence rates were 19.5%, 25.0% and 46.2%, respectively.

#### AFP-L3

AFP-L3 is one of the subtypes of AFP and a specific protein secreted by HCC cells. The recurrence rates in people with AFP-L3 ≥ 5% and < 5% were 21.4–29.3% and 10.0–14.7% at 1 year, and 59.5–64.4% and 33.6–43.5% at 3 years, respectively [19]. The recurrence and metastasis rates were significantly increased in those with AFP-L3 ≥ 10%. The recurrence risk in patients failing to achieve normalization after treatment was five times than that in patients achieving normalization [20].

#### DCP/PIVKA II

DCP is significantly correlated with MVI, recurrence and prognosis of HCC. A meta-analysis of 5647 HCC patients who received curative treatment showed that with 40 or 100 mAU/mL as the cut-off value, the RFS was significantly shortened in patients with high DCP level before radiofrequency ablation (RFA) [21]. The 5-year recurrence rate of 470 HCC patients with DCP ≥ 400 mAU/mL was about two times of that in patients with DCP < 400 mAU/mL before liver resection [22].

#### Other markers

Positive circulating tumor cell (CTC) and circulating tumor DNA (ctDNA) are related to HCC satellite nodules, MVI and poor tumor differentiation. The patients with number of epithelial cell adhesion molecule-positive CTCs (EpCAM^+^-CTC^7.5^) ≥ 2 before liver resection were mostly complicated with satellite lesions, MVI, poorly tumor differentiation and high AFP level, with risk of recurrence increased to 5.2 times compared with those with EpCAM^+^-CTC^7.5^ < 2 [23]. Studies have shown that for patients without HCC recurrence, the positive rate of ctDNA before treatment was significantly lower than that in those with HCC recurrence [24].

### Impact of curative treatment regimens

Curative treatment options of HCC mainly include liver resection, local ablation and liver transplantation, and for some small liver cancers, stereotactic body radiation (SBRT) either alone or in combination with transcatheter arterial chemoembolization (TACE) can also obtain curative treatment effect.

For the selection of liver resection for HCC, it is needed to take into account factors such as tumor volume, location, degree of cirrhosis, liver reserve function and estimated postoperative residual liver volume. Except for tumor factors, the risks of HCC recurrence may be associated with non-anatomical liver resection [25], narrow resection margin [26], intraoperative bleeding and large amount of blood transfusion [27], intraoperative extrusion of tumor, postoperative infection [28] and liver failure. Liver transplantation can be selected for patients with BCLC 0–B or CNLC Ia–IIb HCC with decompensated liver function, unsuitable for surgical resection and local ablation. The recurrence rate after liver resection was significantly higher than that after liver transplantation [29]. A global multicenter study showed that the recurrence rate was 14.1% at 6 years of follow-up after liver transplantation in 1218 HCC patients and 54.4% at 5.6 years of follow-up after liver resection in 2068 HCC patients. A 5-year follow-up study in China included 2796 cases of HCC having undergone liver resection or liver transplantation, in which the recurrence rates were 47.6% and 13.9% in patients who met the Milan criteria [30].

Local ablation includes microwave ablation (MWA), RFA, absolute ethanol injection and cryoablation. In a Chinese report, the IHR rates after MWA was 2.69 times as that after liver resection [31]. In South Korea, among 283 cases of HCC with a paravascular tumor diameter ≤ 3 cm, the cumulative IHR rate at 5 and 10 years after RFA was approximately 1.5–2.5 times as that after liver resection. A number of studies and meta-analyses confirmed that at 1–5 years, there was no significant difference in local recurrence rates between MWA and RFA, nor between RFA and cryoablation for HCC. Studies have reported that for HCC patients with 1–2 tumor nodules and maximum tumor diameter of ≤ 5 cm, the recurrence rate after treatment with SBRT was similar to that after liver resection [32]. In an Asian multicenter study with 2064 HCC patients, the local recurrence rates at 3 years after SBRT and RFA treatment were 21.2% and 27.9%, respectively, and SBRT treatment significantly reduced the recurrence rate in those with tumor diameter ≤ 3 cm or > 3 cm in a subphrenic location [33].

### Etiologically related disorders of HCC

#### Hepatic fibrosis and cirrhosis

Hepatic cirrhosis is an independent risk factor for long-term recurrence of HCC, and the risk of recurrence is 1.5–2.5 times that of non-liver cirrhosis. The long-term recurrence risk in those with liver stiffness measurement (LSM) ≥ 12 kPa is two times than LSM < 12 kPa [34]. A prospective study reported [35] that the median LSM was 11.8, 12.4 and 18.2 kPa, respectively, in those with no recurrence, short-term recurrence and long-term recurrence. The short-term recurrence rate was 3%, 4.6%, 30.3% and 62.1% in those with METAVIR F1–4, respectively. The long-term recurrence was only observed in METAVIR F3 and F4 population, which accounted for 14.8% and 85.2%, respectively. The long-term recurrence rate of Laennec score F4B/4C was up to 2.8 times that of F0–4A patients (66.3% vs 23.5%) [36].

Liver functional reserve in patients with liver cirrhosis reflects the ability of liver metabolism, repair and regeneration. A Chinese study reported that the recurrence rate after curative treatment was 26.8% in Child Pugh A and B patients with HBV-HCC tumor diameter ≤ 3 cm, and 55.6% in Child Pugh C patients [37]. The model for end-stage liver disease (MELD) score before liver transplantation is a reliable method to assess the risk of posttransplantation recurrence [38]. A study in Taiwan showed that the recurrence rate of HCC after liver resection tended to increase with the increase of baseline albumin–bilirubin (ALBI) grade, and the recurrence risk of HCC with ALBI 2–3 was 1.257 times as that with ALBI 1 [39]. Indocyanine green retention rate at 15 min (ICG R15) is often used to evaluate the liver functional reserve and the tolerable volume to be resected before surgical liver resection. It has been reported that after liver resection, the recurrence rate of HCC in patients with ICG R15 > 10% was significantly higher than that in patients with ICG R15 < 10%.

#### Chronic HBV, HCV infection

HBV, HCV infection and high viral load are related to HCC microenvironment and MVI, which are the main risk factors for recurrence of HCC after curative treatment. The MVI risk of HBV–HCC patients with HBV DNA ≥ 2000 IU/mL is 1.399 times that of patients with HBV DNA load < 2000 IU/mL [40]. The recurrence risk in patients with HBV DNA ≥ 100 IU/mL was 2.943 times that in those with HBV DNA < 100 IU/mL [41]. Patients with antiviral treatment ≥ 3 months before curative treatment was observed with the risk of MVI reduced by 40% [42] and the risk of HCC recurrence reduced by 25% [40], and the risk of recurrence was reduced by 45–66% in patients with antiviral treatment ≥ 1 year [43].

The annual recurrence rate of HCV–HCC is increased by 2–5% compared with HBV–HCC [44]. There was no statistically significant difference in the short-term recurrence rate between the population which achieved sustained virological response (SVR) and the population which failed to achieve SVR (36.3% vs 54.3%), but achieving SVR significantly reduced the long-term recurrence rate (32.3% vs 72.9%) and the risk of HCC recurrence (RR = 0.31) [45].

#### Non-infectious chronic liver disease

The 5-year recurrence rate of HCC associated with alcoholic liver disease (ALD), non-alcoholic fatty liver disease (NAFLD), autoimmune liver disease and inherited metabolic liver disease after curative treatment is significantly lower than that of HBV–HCC and HCV–HCC. A Japanese study reported that the 5-year RFS rates after liver resection were 47%, 41%, and 31% in 2738 cases of non-HBV- and non-HCV-related HCC, 2194 cases of HBV–HCC, and 7018 cases of HCV-HCC, respectively. The 5-year DFS rates after liver resection for mild and severe ALD-related HCC were 51.2% and 25.2%, respectively.

### Obesity and diabetes mellitus

Obesity and diabetes mellitus (DM) increase the risk of HCC recurrence. Patients with body mass index (BMI) > 30 kg/m^2^ displayed a 4-fold increased risk for developing MVI and a risk of recurrence 1.9 times [46] after liver transplantation compared with those with BMI ≤ 30 kg/m^2^. Patients with sarcopenic obesity had significantly shorter median RFS than those who are non-sarcopenic non-obesity (8.4 vs 21.4 months), with the hazard ratio (HR) for HCC recurrence of 2.031: 1 [47]. HBV–HCC patients with DM had a risk of MVI 1.69 times as that in those without DM, and patients with DM had a significantly increased 1-year recurrence rate (51.6% vs. 38.3%) [48]. After MWA, the risk of recurrence in patients with fasting blood glucose > 7.0 μmol/L was 2.728 times that in those with normal blood glucose [49]. Median RFS after curative treatment was 13 and 26 months in patients with glycosylated hemoglobin levels > 7.0% and ≤ 7.0%, respectively.

### Other risk factors

Male gender and family history of HCC are risk factors for long-term recurrence of HCC after curative treatment. In a retrospective study of 734 patients in China, the long-term recurrence risk after liver resection in males was 1.372 times as that in females. After RFA, male patients had a risk of long-term recurrence 3.177 times as that observed with females [50]. It is reported in Shanghai, China that among 1,112 cases of HBV–HCC, 183 had a first-degree family history of HCC, and the overall recurrence rate (75.4% vs 53.6%) and long-term recurrence rate (35.2% vs 19.0%) after treatment were significantly higher than those without family history.

- Recommendation 2: HCC tumor diameter > 5 cm, number ≥ 3, and absence of intact capsule, poorly differentiated tumor cells and MVI on pathological observation, with satellite lesions are risk factors for postoperative recurrence (A, 1).
- Recommendation 3: High levels of serum AFP and/or AFP-L3 and DCP before curative treatment are risk factors for HCC recurrence (A, 1).
- Recommendation 4: The risk of HCC recurrence after liver transplantation is lower than liver resection, and the risk of local recurrence after ablation is higher than liver resection (A, 1). Non-anatomic liver resection, narrow resection margin, intraoperative bleeding and large amount of blood transfusion, intraoperative extrusion of tumor and postoperative complications of infection and liver failure are risk factors for HCC recurrence after curative therapy (B, 2).
- Recommendation 5: Chronic HBV or HCV infection and liver cirrhosis are risk factors for HCC recurrence (A, 1). Male gender, family history of HCC, diabetes mellitus, obesity and alcohol consumption increase the risk of HCC recurrence (B, 2).

## Tertiary prevention measures for HCC

### Risk stratification and surveillance for recurrence after curative treatment of HCC

#### Risk stratification for recurrence after curative treatment of HCC

Based on the evidence of HCC recurrence referring to BCLC and CNLC staging, combined related risk factors, this *Consensus* divides the population after curative treatment of HCC into low-, medium-, high- and very high-risk groups of recurrence. The estimated risks of recurrence for each category are < 20%, 20–35%, 35–45% and > 45%.

*Low-risk population*: single tumor diameter ≤ 3 cm (BCLC 0–A/CNLC Ia), with any of the following etiologically related liver diseases: ① low HBV DNA load or achieved virologic response for HBV-HCC patients; ② HCV-HCC patients achieved SVR by antiviral therapy; ③ HCC associated non-infected liver diseases, such as ALD, NAFLD or autoimmune liver disease, etc.

*Medium-risk population*: single tumor diameter ≤ 5 cm (BCLC 0–A/CNLC Ia), with ≥ 1 of the following risk factors: ① HBV-HCC or HCV-HCC with HBV DNA or HCV RNA high load; ② advanced liver fibrosis; ③ family history of liver cancer; ④ diabetes mellitus and/or obesity; ⑤ chronic alcohol consumption.

*High-risk population*: single tumor diameter > 5 cm or 2–3 tumor nodules, the maximum tumor diameter ≤ 3 cm (BCLC A/CNLC Ib), with any of the following risk factors: ① hepatic cirrhosis; ② accompanied by ≥ 1 of the following serological changes: AFP 200–400 ng/mL, AFP-L3 5–10% and DCP 100–400 mAU/mL.

*Very high-risk population*: (1) single tumor diameter > 5 cm or 2–3 tumor nodules, the maximum tumor diameter ≤ 3 cm (BCLC A/CNLC Ib), with ≥ 1 of the following serological changes: AFP ≥ 400 ng/mL; AFP-L3 ≥ 10%; DCP ≥ 400 mAU/mL; (2) 2–3 tumor nodules, with the maximum tumor diameter > 3 cm (CNLC IIa); (3) with ≥ 1 of the following tumor characteristics on liver histopathology: MVI, satellite lesions, and poorly differentiated HCC cells.

Several studies have reported on prediction models of HCC recurrence risk, such as ERASL model pre- and post-liver resection (preERASL, postERASL). The models were established based on gender, ALBI grade, serum AFP level, tumor volume and number, and divided HCC recurrence risk into low, medium and high [12]. Models preMORAL (NLR, AFP, maximum tumor diameter) and postMORAL (tumor grade, MVI, maximum tumor diameter and count) predict the risk of recurrence after transplantation and divide HCC recurrence risk into low, medium, high, and very high [51]. In addition, the RETREAT score (MVI, AFP, maximum tumor diameter, tumor count) [52] assesses the risk of recurrence after transplantation and the AS score (age and international normalized ratio) [53] assesses the risk of recurrence after liver resection and RFA have been documented. However, all of the above models have not yet adopted in clinical application.

#### Surveillance protocol for HCC recurrence

At present, there is no uniform standard surveillance protocol for HCC recurrence internationally. The *2017 National Comprehensive Cancer Network (NCCN) guidelines* recommend surveillance with AFP and imaging every 3–6 months for first 2 years after curative resection of HCC and every 6–12 months thereafter [54]. The *2018 European Society of Medical Oncology (ESMO) guidelines* recommend surveillance with contrast-enhanced CT or MRI every 3 months for first 2 years and every 6 months thereafter [55]. The study in Hong Kong suggested that CT should be performed every 3–4 months for first 2 years and 6–12 months thereafter for individuals with medium or low recurrence risk [56]. It is reported in Guangzhou that CT and/or MRI once every 2.6–3.0 months within 18 months after curative treatment of BCLC B HCC can detect recurrence earlier [57].

**Fig. 2:**
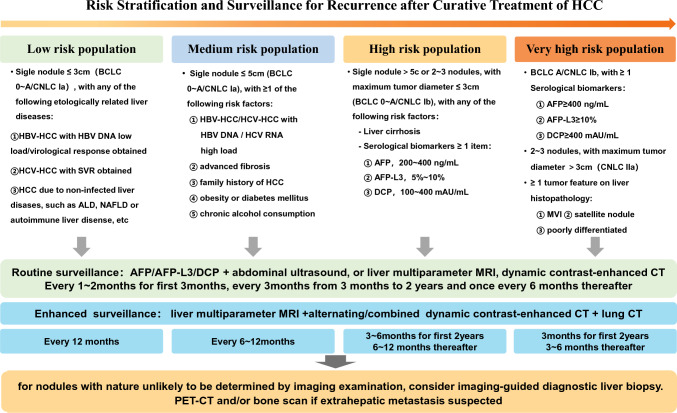
Risk stratification and surveillance for recurrence after curative treatment of HCC. *BCLC* Barcelona clinic liver cancer staging; *CNLC* China liver cancer staging; *SVR* sustained virological response; *ALD* alcoholic liver disease; *NAFLD* non-alcoholic fatty liver disease; *AFP* alpha-fetoprotein; *DCP* des-gamma-carboxy prothrombin; *MVI* microvascular invasion; *MRI* magnetic resonance imaging; *CT* computed tomography imaging; *PET* positron emission tomography

It is recommended to perform dynamic contrast-enhanced CT, MRI or contrast-enhanced ultrasound at 1–2 months after radical treatment to evaluate the treatment effect. Stratified by HCC recurrence risk after curative treatment, serum AFP or combined with AFP-L3, DCP and routine abdominal ultrasound, or liver multiparametric MRI, CT dynamic contrast-enhanced imaging are routinely used to surveil HCC recurrence once every 1–2 months for first 3 months, once every 3 months from 3 months to 2 years and once every 6 months thereafter. For strengthening the surveillance, liver multiparametric MRI or multi-phase dynamic contrast-enhanced CT, simultaneously with lung CT was adopted. When necessary, PET-CT and/or bone scan can be combined, with the surveillance interval of 12 months for individuals with low recurrence risk, 6–12 months for individuals with medium recurrence risk, 3–6 months for first 2 years and 6–12 months thereafter for individuals with high recurrence risk, and 3 months for first 2 years and 3–6 months thereafter for individuals with very high recurrence risk. Diagnostic liver biopsy is considered for new intrahepatic nodules difficult to be determined by imaging examination. PET-CT and/or bone scan can be performed in patients with suspected extrahepatic metastasis involving bones, lymph nodes and multiple organs (Fig. 2).

### Surveillance and diagnosis for HCC recurrence

Methods for surveillance and diagnosis of HCC intrahepatic recurrence can refer to *Consensus on Secondary Prevention of Primary Liver Cancer* [3]. HCC serum marker levels, imaging changes in the liver and extrahepatically involved organs can be used to assess HCC intrahepatic recurrence and extrahepatic metastasis, combined with histopathological examination of tumors when necessary.

#### Serological markers

AFP is the preferred serological marker for screening and surveillance of HCC recurrence. The median time from AFP elevation after curative resection to HCC recurrence detected by imaging was 20 months. For patients with AFP elevated to > 20 ng/mL after curative treatment but negative imaging, the cumulative incidence of HCC recurrence suggested by imaging after 6 months and 1 year was 24.4% and 40.1%, respectively [58]. For patients with decreased AFP after 8 weeks of curative treatment of HCC based on hepatitis/cirrhosis but without normalization, AFP-L3 decreased to < 10% indicates effective treatment. AFP, AFP-L3 and DCP/PIVKA II can be used for surveillance in combination. It has been reported that the sensitivity and specificity of AFP combined with DCP in the diagnosis of recurrent HCC after liver transplantation increased from 59.2% and 88.8% to 92.5%, respectively [59].

#### Imaging examination

(1) *Monitoring of intrahepatic recurrence*: Common imaging methods include routine abdominal ultrasound, contrast-enhanced ultrasonography (CEUS), CT and MRI examination of the liver.

① *Abdominal ultrasound and CEUS*: for assessing the primary liver disease, and surveillance and detection of recurrent tumor diameter > 2 cm. Rapid enhancement in the arterial phase of CEUS with elevated serum AFP had a sensitivity of 97% and a specificity of 68% in the diagnosis of HCC recurrence [60].

② *Liver CT*: Liver CT had a sensitivity of 54% and a specificity of 92% in the diagnosis of intrahepatic HCC recurrence ≤ 2 cm [61]. Both of the sensitivity and specificity of dynamic contrast-enhanced CT are 72% for diagnosis of HCC intrahepatic recurrence tumor ≤ 3 cm [60].

③ *Liver multiparametric MRI*: For the preferred imaging method to evaluate the effect of curative treatment and enhanced surveillance of HCC recurrence, gadolinium ethoxybenzyl diethylenetriamine pentaacetic acid (Gd-EOB-DTPA)-enhanced MRI had a sensitivity of 69–88% and a specificity of 73–94% in the diagnosis of HCC intrahepatic recurrence tumor size ≤ 2 cm [61, 62]. It has been reported that about 80–95% of liver nodules ≤ 1 cm with typical HCC features on Gd-EOB-DTPA-enhanced MRI after curative treatment were recurrent HCC [63, 64]. Gd-EOB-DTPA-enhanced MRI alternating or combined with dynamic contrast-enhanced CT can detect HCC recurrence earlier.

④ *Histopathological examination of liver*: For hepatic space-occupying lesions lacking typical HCC imaging features, histopathological examination helps to clarify the nature of the lesion, confirm the diagnosis, or rule out HCC recurrence. Nevertheless, the potential risks should be considered before liver biopsy, predominantly the morbidity of pain, bleeding and needle track seeding of tumor [65].

(2) *Monitoring of HCC extrahepatic metastasis*: Common sites of HCC extrahepatic metastasis include lung (39–55%), lymph nodes (34–53%), bone (2–39%), adrenal gland (1.2–21%), etc. [66–68]. Lung metastasis is more common in the lower lobe and is mainly characterized by non-calcified soft tissue nodules. Abdominal lymph node metastasis is common in perihepatic, peripancreatic and retroperitoneal lymph nodes. Abdominal ultrasound and contrast-enhanced CT shows enlarged lymph nodes, characterized by atypia, arterial phase enhancement and central necrosis. The predilection sites of bone metastasis are spine, pelvis, ribs, sternum, head, etc., and the imaging of CT, MRI, bone scan and PET-CT is characterized by osteolytic bone destruction and soft tissue mass. PET-CT had a sensitivity of 64–77% and a specificity of 95–98% in the diagnosis of extrahepatic lymph node, bone and other metastasis [69, 70]. Adrenal metastasis has malignant tumor findings such as heterogeneous enhancement of density and ill-defined borders on contrast-enhanced CT.

- Recommendation 6: For routine surveillance of HCC recurrence, serum AFP and/or AFP-L3, DCP combined with abdominal ultrasound, or multiparametric MRI, dynamic contrast-enhanced CT can be adopted to surveil HCC intrahepatic recurrence; for enhanced surveillance of HCC recurrence, multiparametric MRI alternating or combined with dynamic contrast-enhanced CT can be used based on serological surveillance to surveil intrahepatic recurrence and extrahepatic metastasis, combined with PET-CT and/or bone scan when necessary (A, 1).
- Recommendation 7: After curative treatment of HCC, routine surveillance should be performed once every 1–2 months for first 3 months, once every 3 months from 3 months to 2 years and once every 6 months thereafter. Enhanced surveillance can be performed every 12 months for individuals with low risk, every 6–12 months for individuals with medium risk, every 3–6 months for first 2 years and 6–12 months thereafter for individuals with high risk, and every 3 months for first 2 years and every 3–6 months thereafter for individuals with very high risk (B, 1).
- Recommendation 8: For suspected nodules detected during routine surveillance or accompanied by serum AFP > 20 ng/mL and/or AFP-L3 > 10%, DCP > 40 mAU/mL, an enhanced surveillance procedure for HCC recurrence should be initiated. Hepatocyte-specific contrast medium Gd-EOB-DTPA-enhanced MRI is feasible for suspected nodules ≤ 1 cm in diameter (A, 1). For intrahepatic nodules with nature unlikely to be determined by imaging examination, consider imaging-guided diagnostic liver biopsy (C, 1). PET-CT and/or bone scan can be performed in patients with suspected extrahepatic metastasis involving bones, lymph nodes and multiple organs (B, 1).

### Treatment for etiologically related diseases of HCC

#### Antiviral therapy for chronic HBV and HCV infection

By referring to *East Asia expert opinion on treatment initiation for chronic hepatitis B* [71] and *APASL consensus statements and recommendation on treatment of hepatitis C* [72] and *Expert Consensus on Antiviral Therapy for HBV/HCV-related Hepatocellular Carcinoma: a 2021 Update* [73], antiviral therapy with first-line nucleos(t)ide analogues (NAs) (entecavir, tenofovir, tenofovir alafenamide) can be applied after curative treatment of HBV–HCC, and antiviral therapy with pegylated interferon α (PEG-IFNα) can be considered for patients without contraindications. Patients with HCV–HCC can be treated with DAAs to achieve SVR.

Several randomized controlled studies have confirmed that the application of NAs and/or interferon therapy after curative treatment of HBV-related HCC can prolong RFS regardless of viral load [43, 74]. In a global multicenter retrospective study with 642 patients with HCV–HCC, the recurrence rates of HCC in patients treated with DAAs, interferon, or no antiviral therapy were 6.3%, 11.4%, and 28.2% after liver transplantation, respectively [75]. An Italian study included 491 cases of BCLC 0/A HCV-HCC treated with curative treatment, the HCC recurrence rate was reduced by DAAs treatment and achievement of SVR [76].

#### Treatment for other liver and systemic diseases

Alcohol withdrawal improves physical activity scores and survival in patients with ALD-related HCC after liver resection [77]. Strict control of glycosylated hemoglobin ≤ 9% in patients with DM can reduce the risk of HCC recurrence [78], and hypoglycemic therapy with metformin can prolong RFS after curative treatment [79].

- Recommendation 9: After curative treatment of HBV-HCC, patients with positive HBsAg and/or HBV DNA can be treated with first-line NAs, and PEG-IFNα can be considered for patients without contraindications (A, 1); Patients with HCV-HCC with positive HCV RNA can be treated with DAAs to achieve SVR and reduce the risk of recurrence(B, 1).
- Recommendation 10: Patients with ALD-related HCC should strictly abstain from alcohol after curative treatment (B, 1).
- Recommendation 11: Patients with HCC accompanied by diabetes mellitus and obesity should have strict control of blood glucose and body weight after curative treatment (B, 1).

### Treatment against recurrence after curative treatment of HCC

#### TACE

It is an important treatment against tumor recurrence after curative treatment of HCC. Studies have shown that adjuvant TACE after curative treatment can improve RFS in HCC patients with moderate or high risk of recurrence [80, 81]. In a Phase III, randomized, controlled study conducted by Zhongshan Hospital, China, adjuvant TACE improved RFS in HBV–HCC with moderate (single tumor > 5 cm without MVI) or high (single tumor with MVI or 2–3 tumors) risk of recurrence after liver resection [80]. A meta-analysis showed that in a total of 12 studies with 2190 HCC patients with MVI, especially for those with tumor diameter > 5 cm or multinodular tumors, liver resection with adjuvant TACE reduced the 5-year recurrence rate and improved the 5-year overall survival rate compared with liver resection alone [81].

#### Hepatic artery infusion chemotherapy (HAIC)

HAIC treatment can increase the local drug concentration in the liver, and reduce the distribution of chemotherapeutic drugs in other organs, with strong anti-tumor effect and less systemic side effects. Adjuvant HAIC treatment after liver resection for HCC has been shown to significantly improve the 5-year RFS rate, reduce the risk of intrahepatic recurrence by 44% [82], and reduce the intrahepatic recurrence rate by 12.1% [83]. A meta-analysis showed that adjuvant HAIC treatment after liver resection for HCC improved DFS compared with liver resection alone (HR approximately 1: 0.6) [84].

#### Radiotherapy (RT)

External radiotherapy includes three-dimensional conformal radiation therapy (3D-CRT), stereotactic body radiation therapy (SBRT) [85] and intensity modulated radiation therapy (IMRT) [86, 87], etc., and internal radiotherapy includes radioactive iodine, ^131^I monoclonal antibody, liver section ^125^I particle implantation, etc. [88]. Local radiotherapy for surgical margins may reduce the local recurrence rate of HCC with narrow margins and MVI.

#### Molecular targeted therapy

It is reported that for HCC with MVI, adjuvant sorafenib after curative treatment significantly reduced long-term recurrence rate (43.7% vs 75.8%) [89]. The STORM study included 1114 cases of HCC in 202 hospitals across 28 countries, and the results showed that the application of sorafenib failed to significantly prolong TTR and RFS. In two meta-analyses, 1545 cases and 2655 cases of HCC were included, respectively, and the recurrence rate was significantly reduced in the adjuvant sorafenib treatment population after curative liver resection [90, 91]. Therefore, the population which can benefit from the anti-angiogenic drugs combined with TACE adjuvant therapy, is still needed to be further investigated.

#### Immunotherapy

These include adoptive immunotherapy, tumor vaccines, immune checkpoint inhibitors (ICIs), and immunomodulatory agents. A meta-analysis displayed that adjuvant adoptive immunotherapy or combined dendritic cell vaccines can reduce the recurrence rate after curative treatment of HCC [92, 93]. In recent years, a number of clinical studies of ICIs either alone or combined with molecular targeted drugs to prevent recurrence after curative resection of HCC are ongoing, such as CheckMate-9DX, keynot937, IMbrave050, and EMERALD-2. The IMbrave 050 is a phase 3 study of adjuvant atezolizumab plus bevacizumab versus active surveillance in patients with HCC at high risk of disease recurrence following resection or ablation. At the prespecified interim analysis (the data was recently presented in the Annual meeting of American Association of Cancer Research, AARC 2023), adjuvant atezolizumab and bevacizumab significantly improved RFS and reduced the recurrence risk by 28%, compared with active surveillance group, at a median follow-up of 17.4 months.

The adjustment of immunosuppressive agents after liver transplantation helps to reduce the risk of postoperative HCC recurrence, and low-dose calcineurin inhibitor (CNI, such as tacrolimus, cyclosporine A, etc.) and glucocorticoid early withdrawal regimens can reduce HCC recurrence rates [94]. A meta-analysis showed that regimens based on mammalian target of rapamycin inhibitors (sirolimus and everolimus) were associated with significantly lower recurrence rates than CNI treatment [95].

- Recommendation 12: For HCC with MVI, tumor diameter > 5 cm or multiple nodules, adjuvant TACE can be adopted after liver resection to reduce the risk of recurrence (A, 1).
- Recommendation 13: Liver resection for HCC with narrow resection margin and MVI may be followed by adjuvant external radiotherapy or ^125^I particle internal radiotherapy to reduce or delay HCC recurrence (B, 2).
- Recommendation 14: Adjuvant molecular targeted drugs such as sorafenib or combined TACE may be considered after curative treatment of HCC, but it is still needed to further clarify the population which can benefit from the treatment (B, 2).
- Recommendation 15: Adjuvant treatment with immune checkpoint inhibitors and vascular endothelial growth factor inhibitors, such as atezolizumab plus bevacizumab, might be considered for early-stage HCC at high risk recurrence after liver resection or ablation to reduce or delay HCC recurrence (B, 2).

## Clinical issues to be studied and addressed

1. Relationship between biological characteristics, epigenetics and recurrence of HCC.
2. Relationship between host gene polymorphisms, virus and host gene integration and HCC recurrence.
3. Serological markers with high sensitivity and specificity for surveillance and early diagnosis of HCC recurrence.
4. Individualized regimens for the prevention of HCC recurrence.
5. Effect and prospect of neoadjuvant therapy such as targeted drugs and/or immune checkpoint inhibitors in preventing HCC recurrence.
6. Effect of neoadjuvant anti-tumor antigen vaccine in preventing recurrence after curative treatment of HCC.
7. Health economics of tertiary prevention measures for primary liver cancer.

## Data Availability

Data is available on request.

## Abbreviations

HCC: Hepatocellular carcinoma
ICC: Intrahepatic cholangiocarcinoma
BCLC: Barcelona clinic liver cancer staging
CNLC: China liver cancer staging
IHR: Intrahepatic recurrence
MVI: Microvascular invasion
IM: Intrahepatic metastasis
MO: Multicentric occurrence
EHM: Extrahepatic metastasis
RFS: Recurrence-free survival
TTR: Time to recurrence
AFP: Alpha-fetoprotein
DCP: Des-gamma-carboxy prothrombin
PIVKA II: Protein induced by vitamin K absence or antagonist II
AFP-L3: Lens culinaris agglutinin-reactive fraction of AFP
CTC: Circulating tumor cell
ctDNA: Circulating tumor DNA
CEUS: Contrast-enhanced ultrasonography
Gd-EOB-DTPA: Gadolinium ethoxybenzyl diethylenetriamine pentaacetic acid
CT: Computed tomography imaging
MRI: Magnetic resonance imaging
PET: Positron emission tomography
LSM: Liver stiffness measurement
NAs: Nucleoside (acid) analogues
DAA: Direct-acting antiviral drugs
SVR: Sustained virological response
NAFLD: Non-alcoholic fatty liver disease
T2DM: Type 2 diabetes mellitus
ALD: Alcoholic liver disease
HBV: Hepatitis B virus
HCV: Hepatitis C virus
PEG-IFNα: Pegylated interferon α
MWA: Microwave ablation
RFA: Radiofrequency ablation
SBRT: Stereotactic body radiation therapy
TACE: Transcatheter arterial chemoembolization
HAIC: Hepatic artery infusion chemotherapy
ICIs: Immune checkpoint inhibitors
MELD: Model for end-stage liver disease
ALBI: Albumin–bilirubin
TCM: Traditional Chinese medicine
